# Cogena, a novel tool for co-expressed gene-set enrichment analysis, applied to drug repositioning and drug mode of action discovery

**DOI:** 10.1186/s12864-016-2737-8

**Published:** 2016-05-27

**Authors:** Zhilong Jia, Ying Liu, Naiyang Guan, Xiaochen Bo, Zhigang Luo, Michael R. Barnes

**Affiliations:** Department of Chemistry and Biology, College of Science, National University of Defense Technology, Changsha, Hunan 410073 People’s Republic of China; William Harvey Research Institute, Queen Mary University of London, Charterhouse Square, London, EC1M 6BQ UK; Hunan Key Laboratory of Medical Epigenomics, Department of Dermatology, Second Xiangya Hospital, Central South University, Changsha, Hunan 410011 People’s Republic of China; College of Computer, National University of Defense Technology, Changsha, 410073 People’s Republic of China; National Laboratory for Parallel and Distributed Processing, National University of Defense Technology, Changsha, 410073 People’s Republic of China; Beijing Institute of Radiation Medicine, Beijing, 100850 People’s Republic of China

**Keywords:** Drug repositioning, Pathway analysis, Mode of action, Psoriasis

## Abstract

**Background:**

Drug repositioning, finding new indications for existing drugs, has gained much recent attention as a potentially efficient and economical strategy for accelerating new therapies into the clinic. Although improvement in the sensitivity of computational drug repositioning methods has identified numerous credible repositioning opportunities, few have been progressed. Arguably the “black box” nature of drug action in a new indication is one of the main blocks to progression, highlighting the need for methods that inform on the broader target mechanism in the disease context.

**Results:**

We demonstrate that the analysis of co-expressed genes may be a critical first step towards illumination of both disease pathology and mode of drug action. We achieve this using a novel framework, **co**-expressed **g**ene-set enrichment analysis (cogena) for co-expression analysis of gene expression signatures and gene set enrichment analysis of co-expressed genes. The cogena framework enables simultaneous, pathway driven, disease and drug repositioning analysis. Cogena can be used to illuminate coordinated changes within disease transcriptomes and identify drugs acting mechanistically within this framework. We illustrate this using a psoriatic skin transcriptome, as an exemplar, and recover two widely used Psoriasis drugs (Methotrexate and Ciclosporin) with distinct modes of action. Cogena out-performs the results of Connectivity Map and NFFinder webservers in similar disease transcriptome analyses. Furthermore, we investigated the literature support for the other top-ranked compounds to treat psoriasis and showed how the outputs of cogena analysis can contribute new insight to support the progression of drugs into the clinic. We have made cogena freely available within Bioconductor or https://github.com/zhilongjia/cogena.

**Conclusions:**

In conclusion, by targeting co-expressed genes within disease transcriptomes, cogena offers novel biological insight, which can be effectively harnessed for drug discovery and repositioning, allowing the grouping and prioritisation of drug repositioning candidates on the basis of putative mode of action.

**Electronic supplementary material:**

The online version of this article (doi:10.1186/s12864-016-2737-8) contains supplementary material, which is available to authorized users.

## Background

The well documented challenges of novel drug development have made the repositioning (or repurposing) of existing drugs to new indications, an attractive and efficient economic prospect for translation of drugs into the clinic [1–3]. Historically, drug repurposing has often been a serendipitous process during drug development where a previously unrecognized on-target or off-target effect is identified and subsequently developed as a new indication (such as sildenafil [1]). Today a rapidly accumulating public corpus of omics data related to disease mechanism and drug action makes complex drug repositioning quite feasible *in silico*. Public databases such as the NCBI Gene Expression Omnibus (GEO) [4], the Connectivity Map (CMap) project [5] and the Library of Integrated Cellular Signatures (LINCS) project (www.lincsproject.org) [6], make systematic computational drug repositioning a credible proposition. One of the basic hypotheses behind CMap is that the drug-induced gene expression should to some degree inversely correlate with the disease-induced gene expression, thus reversing or antagonising the disease process. Other tools have been developed on a similar premise. For example, NFFinder uses transcriptomic data to discover relationships between drugs, diseases or a phenotype of interest [7]. Cheng et al. identified drug-indication pairs via a new similarity scoring algorithm, XSum, of gene expression profiles [8]. These methods have been very widely applied [9, 10], but are still showing limited translation to the clinic.

We would argue, that the failure to translate lies partly in the “black box” nature of such drug repositioning efforts – at a transcriptome wide level it is difficult to separate cause and effect in a disease process among thousands of differentially expressed transcripts. Thus, this approach does little to disclose the mode of action (MoA) of a drug in a specific disease, much less Mechanism of action (MOA). As the recent struggles of the pharmaceutical industry attest [11], a good understanding of both disease mechanism and drug MoA as well as MOA, and importantly how the two align, is a critical component of the target validation required for successful drug development.

Co-expressed genes often work in concert in biological processes, under tight regulatory control, thus conferring an advantage in adaptive evolution [12]. Good evidence supports this. For example, over 22,000 paired co-expression partners were shown to be profoundly conserved between yeast, worm and human [13]. Subsequent studies by the ENCODE consortium have extended the view of conserved complex co-expression modules further and also identified lineage specific co-expression modules [14]. Genes in co-expression modules have been shown to be involved in the same biological pathways [13] and of high disease prognostic value [15]. Cluster analysis, as the backbone of co-expression analysis, is a powerful strategy for the exploration of expression data in the absence of a-priori hypotheses, using results as a classifier [16]. For example gene sets can be extracted from co-expressed clusters and subjected to gene set enrichment analysis.

We hypothesize that genes which are both differentially expressed and co-regulated in a biological state, are more likely to be drivers of the underlying biology and thus co-expression is a critical layer of information to include in pathway analysis. Usually gene set enrichment analysis is performed on a ranked list of expressed genes, or a subset of differentially expressed (DE) genes based on a statistical threshold. Other tools such as WGCNA [17], Human gene correlation analysis (HGCA) [18], STARNET [19], GeneFriends [20] and CoExpress [21] allow the study of co-expressed genes using a weighted correlation network allowing network construction based on a soft thresholding of the correlation coefficient. Tools such as WGCNA are widely used, but most require the user to export analyses into gene set enrichment tools, such as DAVID [22] and Enrichr [23], or commercial tools such as Ingenuity Pathway Analysis (QIAGEN, Redwood City). We have sought to maximise the utility of cogena, by refining the input to include information both on co-expression and differential expression, using a hard threshold for the latter, and feeding the results directly into gene set enrichment. Thus cogena offers an integrated analysis suite, which we show can be complementary, and often more informative than other approaches.

Pathways can bridge the gaps between diseases and drugs, especially when the knowledge concerning drugs, targets, genes and diseases is scarce or unknown. Bayesian matrix factorisation has been used to identify pathways perturbed by drugs and the inferred pathway correlation has been used to reveal the relationship between the underlying pathways perturbed by drugs and disease pathology [24]. Pathways perturbed by sixteen drugs were evaluated based on their targets and used for exposing the indications of drugs [25]. In another study, a link map between small molecules and pathways was constructed using gene expression profiles of cancer, KEGG pathway and CMap data and molecules that significantly affected the same pathways tended to treat the same diseases [26]. Others have used linear models to infer drug mechanism of action at a pathway level from differentially expressed genes and their predicted targets [27]. Causal inference has also been used in a layered drug-target-pathway-gene-disease network to repurpose drugs using statistical learning [28]. Although the above approaches are effective in the discovery of new indications for drugs, their outputs rarely focus on the core genes linking disease and drug mechanism.

Based upon the above considerations, we propose a novel framework for **co**-expressed **g**ene-set **en**richment **a**nalysis (cogena). We hypothesize that highly correlated events may be of great mechanistic relevance to disease and drug action. Accordingly, we have constructed a drug repositioning and drug MoA discovery pipeline based on the cogena framework using a pathway gene set and the CMap gene set. Other gene sets could easily be incorporated by the user. A psoriatic skin transcriptome dataset is used as an exemplar to show the power of cogena. In this case we recovered two approved drugs for psoriasis with different MoA, and identified several novel drugs with potential for repositioning to this disease. Similar results are obtained by cogena using another dataset. In conclusion, by leveraging co-expressed gene profiles in a disease state, cogena offers a powerful tool for studying disease pathology, which can be used to inform drug repositioning and MoA discovery.

## Implementation

### Overview of cogena

**Fig. 1:**
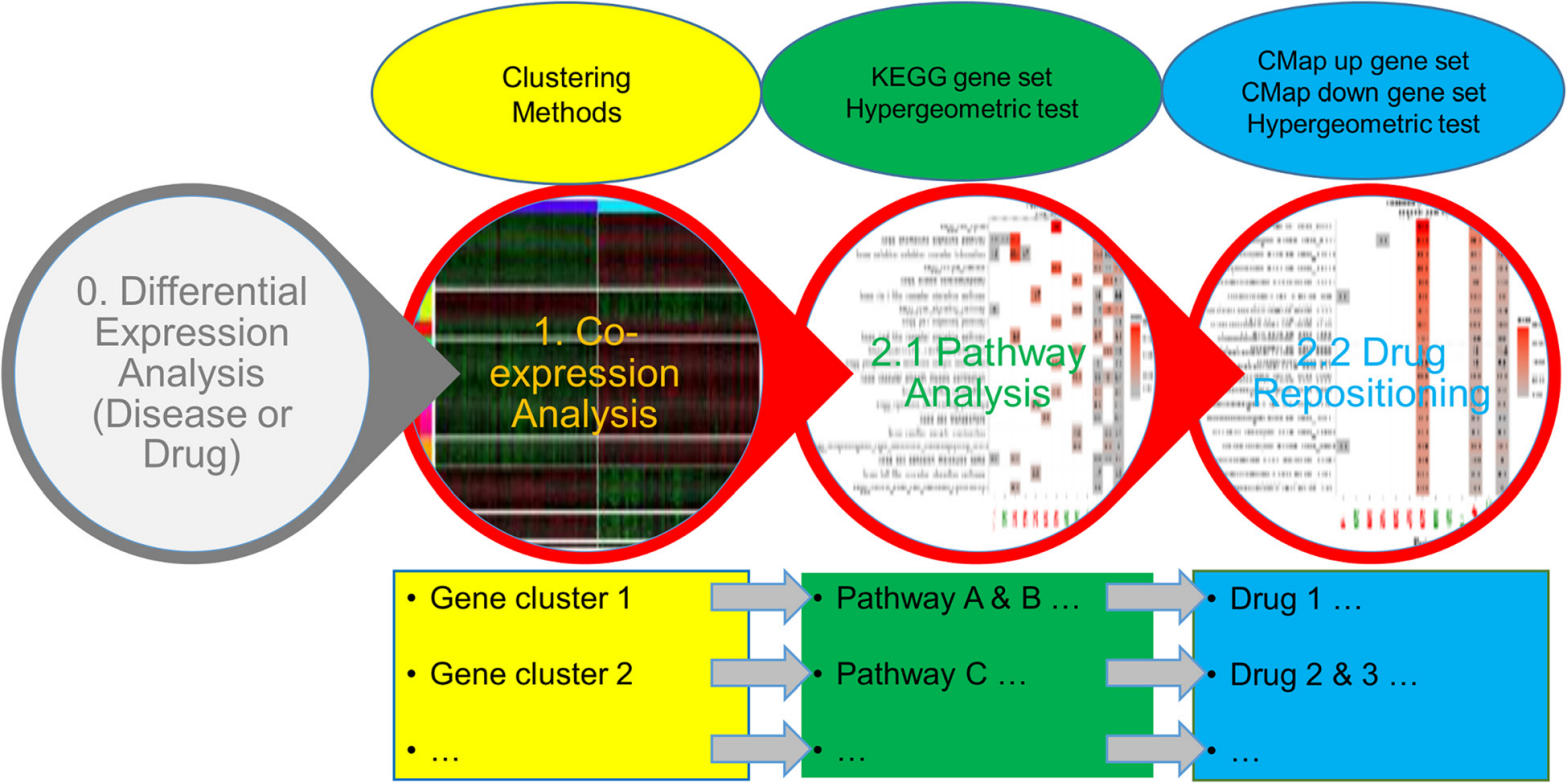
The workflow of cogena for drug repositioning. 0. Differential expression analysis of a disease or compound (drug) dataset. Cogena requires the gene expression signature of differentially expressed genes as an input. 1. Co-expression analysis on the expression profile of differentially expressed genes. Ten clustering methods are available in cogena. 2.1 Pathway Analysis. Hypergeometric test and KEGG gene sets are used for the pathway analysis for each co-expressed gene cluster. 2.2 Drug repositioning. The same test and CMap gene set from Connectivity Map are used for drug repositioning for each co-expressed gene cluster. The pathways enriched for a gene cluster may imply the MoA of drugs enriched for the same gene cluster. The main steps are outlined in the circles, methods in ellipses, while data streams are described in the rectangles

The drug repositioning workflow, implemented within the cogena framework, has three steps (Fig. 1). Firstly, co-expression analysis, using a variety of clustering methods (See below), is performed on the expression of genes showing differential expression in the disease state. Then based on hypergeometric test, pathway analysis and drug repositioning analysis for the co-expressed gene groups are performed respectively. Finally, the putative drug MoA in the disease state can be inferred from the pathway analysis and the known MoA of drug in the same cluster. Collectively, the outputs of the analysis can be used to inform drug discovery and repositioning as discussed below. On the other hand, cogena can identify drugs with similar MoA when the input is drug related gene expression profile. As a tool, cogena has several optional parameters with default values, making it convenient and flexible to use for inexperienced users, with full configurability for advanced users.

### Clustering methods

Cogena leverages various clustering methods based on components of the clValid R package [29] with enhancements for parallel processing. In total there are ten clustering methods available in cogena, Hierarchical Clustering, Agnes, Diana, K-means, Fanny, SOM, Model, SOTA, PAM and CLARA. A detailed description of these methods can be found in the clValid package vignette.

Because all the above clustering methods will request a number to assign the number of clusters to generate, users are able to assign a vector to this parameter, as used in clValid. To speed up the calculation, enhancing the original clValid package, this process is run in parallel with the doParallel package [30].

Different distance metrics have distinct effects upon the clustering methods, therefore we supply a range of metrics. The following distance metrics can be run: Euclidean, Pearson, absolute Pearson, correlation (centred Pearson), absolute correlation, Spearman and biweight correlations (biwt) via the packages amap [31] and biwt [32] respectively.

For distance metric parameters, some clustering methods cannot use all the distance metrics, while Hierarchical Clustering, Diana, Fanny, PAM and Agnes can. K-means can use all except biwt and will change it to correlation automatically. CLARA uses Manhattan or Euclidean, other metrics will be changed as Euclidean. SOTA uses correlation or Euclidean, other metrics will be changed as correlation. The Model method uses its own metric and SOM uses Euclidean only.

As there are no automatic ways to select clustering methods and the number of clusters, the user needs to apply some basic principles to select the most appropriate cluster method and cluster number. For example, an optimal clustering configuration should cluster a specific gene set or pathway across as few clusters as possible, ideally one cluster. Distribution of a gene set across multiple clusters may indicate that too many clusters have been selected for analysis. Unrelated gene sets should be separately enriched among different clusters. Additionally, the number of genes in a cluster should be at a minimum to provide an optimal maximum enrichment score.

### The gene sets

The cogena framework is modular and can be configured to address a range of functions, depending on the gene sets leveraged. For this analysis, we have pre-packaged some of the gene sets from the Molecular Signatures Database (MSigDB), such as the canonical pathways, Gene Ontology (GO) biological process and KEGG gene sets [33]. To launch drug repositioning analysis, we extracted the top 100 and bottom 100 DE genes to generate two gene-set libraries from CMap [5], one for the up-regulated genes and the other for the down-regulated genes for each condition. We selected the top 100 genes, representing the most significant gene group, rather than a statistical threshold to enable consistent comparison across different drugs in similar manner to that employed by other tools, such as Enrichr [23]. Each set is associated with a compound name, cell-type, concentration and instance number, which makes it convenient to merge different instances of a compound based on different conditions. Additionally, cogena can also load user-defined gene sets in Gene Matrix Transposed (gmt) format.

### The hypergeometric test of enrichment analysis

We apply a hypergeometric test for gene set enrichment analysis. The null hypothesis is that there is no relationship between a gene list in a cluster containing *n* genes and a given gene set containing *m* genes. We can model the number of significant genes using a hypergeometric distribution. If there are *k* significant genes in the gene set category, we simply compute the probability of seeing *k* or more significant genes in *n* draws, without replacement, from the reference background gene lists containing *N* genes. Then the probability is given by formula (1). Finally, Benjamini & Hochberg correction for multiple hypothesis testing is applied to the p values.

1 $$ P\left\{x\ge k\right\}={\displaystyle \sum_{x=k}^{\infty}\left(\begin{array}{c}\hfill m\hfill \\ {}\hfill x\hfill \end{array}\right)}\left(\begin{array}{c}\hfill N-m\hfill \\ {}\hfill n-x\hfill \end{array}\right)/\left(\begin{array}{c}\hfill N\hfill \\ {}\hfill n\hfill \end{array}\right) $$

### Visualization of co-expressed gene set enrichment

Heatmaps are used to visualise co-expressed gene set enrichment. Firstly, a gene expression heatmap, with numbered clusters, is used to represent the differential gene expression. The up-regulated and down-regulated genes are identified by a coloured bar on the far left of the heatmap and individual clusters are highlighted by coloured and numbered blocks. The genes contained in the numbered clusters are subjected to gene set enrichment analysis and the results are reported in another heatmap showing the negative log2 false discovery rate (FDR) as an enrichment score for the gene sets. The enrichment scores can be ranked by various conditions, the “mean” and “max” rank by mean or max of the clusters and all DE genes, respectively, while “all” ranks by the enrichment score of all the DE genes. At a more granular level, “up” and “down” rank based on the up- or down-regulated genes respectively. Finally clusters can be ranked by a number *i* representing the *i*-th cluster. It should be noted that the rank of enriched gene sets is likely to be more informative than their absolute scores [34]. Multi-instance CMap drugs with enrichment score above a threshold, such as –log2 (0.05), can be merged based on different conditions (such as cell-type) and visualised by another heatmap.

## Results and Discussion

In the following, we describe an example of co-expression analysis, intra-cluster protein-protein interaction analysis, pathway analysis and drug repositioning analysis using cogena. Finally, putative drug mode of action is illustrated by aligning the pathway analysis and drug repositioning analysis.

### Cogena analysis exemplar

We use a psoriatic skin transcriptome dataset (GSE13355) from NCBI GEO to demonstrate the utility of cogena. Transcriptome expression in psoriasis lesions and non-lesional skin from 58 psoriasis affected individuals was profiled on Affymetrix Human Genome U133 Plus 2.0 microarrays [35]. The raw data were normalised using rma [36] and non-expressed and non-informative genes were filtered using the MetaDE package [37]. The limma package [38] was used to identify DE genes with the thresholds of FDR less than 0.05 and absolute logFC more than 1. All code used to produce the results in this paper are available within https://github.com/zhilongjia/psoriasis.

### Co-expression analysis

Firstly, co-expression analysis was performed using the coExp function. After differential expression analysis of the dataset, cogena used all the implemented clustering methods, using clusters ranging from 2 to 20 and the “correlation” distance metric to analyse DE genes. In the exemplar analysis, the PAM method and 10 clusters were chosen based on these principles and pathway analysis below (See Implementation and Additional file 1: Figure S1).

### Co-expressed genes are enriched for intra-cluster interactions

Cogena was developed on the assumption that co-expressed genes may interact biologically more at an intra-cluster level than an inter-cluster level. We specifically investigated the extent of protein-protein interactions among genes in clusters based on a protein-protein interaction database, STRING [39] (See Table 1). The expected interaction and p value are calculated based on a random background model that preserves the degree distribution of the input proteins [39, 40], implemented via the get_summary function in the STRINGdb package.

**Table 1:** Summary of interactions within clusters for GSE13355

	#gene in cluster	#protein in STRING	#interaction	#expected interaction	Ratio (#interaction/#expected interaction)	p value
Cluster 1	158	152	109	42	2.60	0
Cluster 2	65	62	15	7	2.14	0.0079
Cluster 3	38	36	72	8	9	0
Cluster 4	92	91	19	15	1.27	0.25
Cluster 5	50	49	255	11	23.18	0
Cluster 6	67	65	22	5	4.40	0.0000002
Cluster 7	63	61	463	40	11.58	0
Cluster 8	94	90	19	11	1.73	0.034
Cluster 9	61	61	59	16	3.69	0
Cluster 10	18	18	3	0	NA	0.0048
Up	468	453	1616	633	2.55	0
Down	238	231	235	112	2.10	0
All DE genes	706	684	2407	1274	1.89	0

STRING interactions are shown for each cluster, up or down-regulated genes and all DE genes, how many genes (#gene in cluster), how many proteins (#protein in STRING), how many interactions (#interaction), how many expected interactions (#expected interaction), the ratio of #interactions and #expected interactions, together with the p value to get such a number of interactions by chance

The results demonstrate that co-expressed genes in cluster 1, 3, 5, 7, 9, up or down-regulated genes and all DE genes are highly connected, while genes in other clusters are less connected. Furthermore, based on the ratio of actual interaction and expected interaction, the connectivity between genes in cluster 3 (with ratio value 9), 5 (23.18), 7 (11.58) and 9 (3.69) is higher than those in other clusters. Consequently, we propose that clusters with such properties may be more biologically relevant to the disease phenotype and consequently more tractable to drug intervention.

### KEGG pathway analysis

After co-expression analysis, pathway analysis was performed using the clEnrich function based on KEGG gene sets. The pathogenesis of psoriasis resembles in many aspects an adaptive immune reaction that initiates an abnormal regenerative response of the skin leading to plaque formation [41]. The results of pathway analysis obtained with cogena were compared with Gene Set Enrichment Analysis (GSEA) [42] and WGCNA. For GSEA, the RMA-normalised expression data were queried using the default parameters. For WGCNA, all expressed genes were used with the default parameters and soft thresholds (power) equal 7 based on the scale-free topology fit indices. For the purposes of direct comparison of cogena and WGCNA, co-expressed genes obtained from WGCNA were fed into the cogena pipeline using custom scripts.

**Fig. 2:**
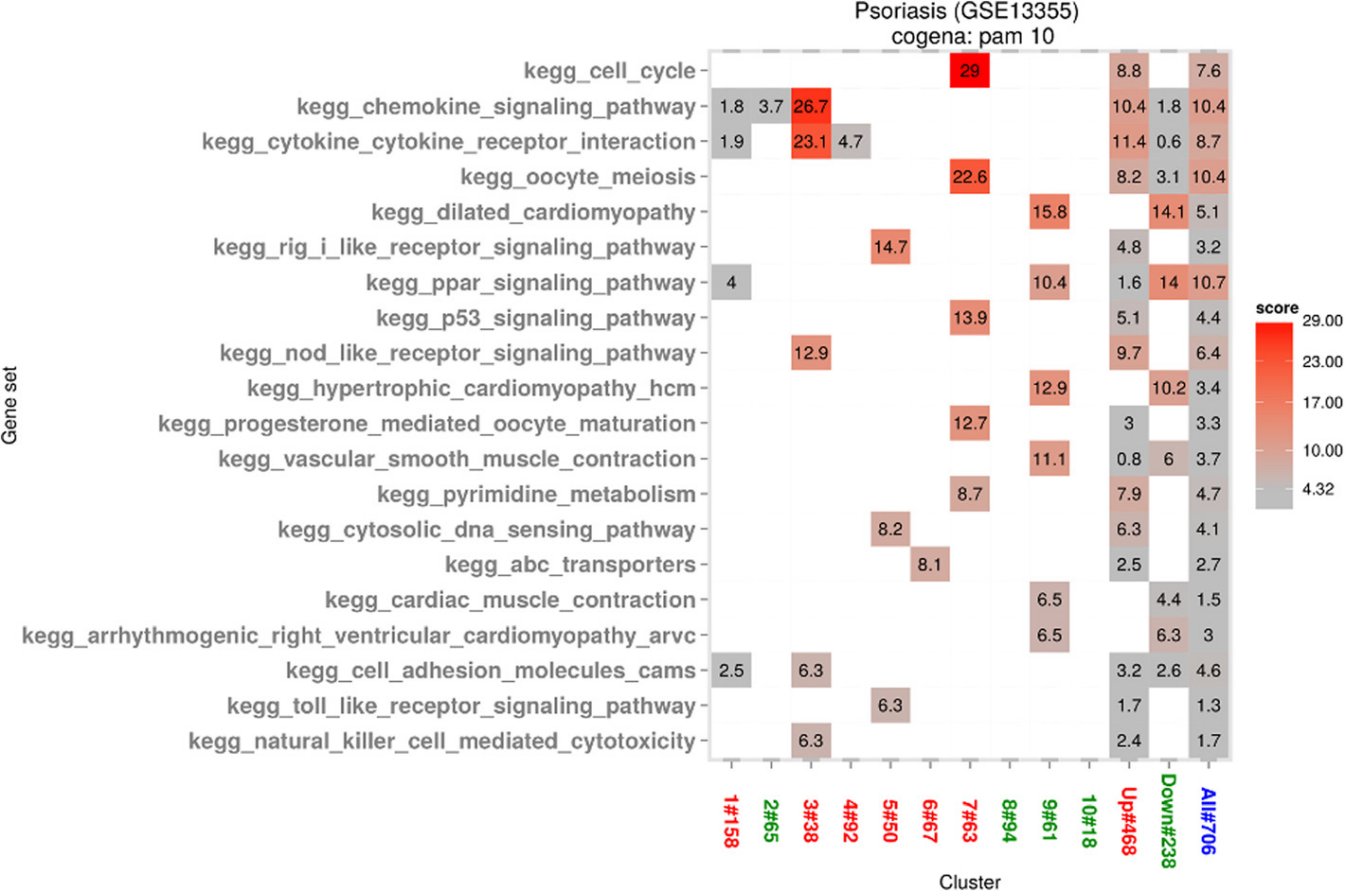
KEGG pathway analysis results by cogena for GSE13355. The enrichment scores are shown based on different clusters, up-regulated, down-regulated and DE genes. And the score is correlated with the depth of colour. In the x axis, the up-regulated clusters are coloured red, while down-regulated clusters are coloured green and cluster containing all DE genes is coloured blue. The ranked pathways are shown in the y axis

We compared the top 20 enriched pathways identified by cogena, WGCNA and GSEA (Fig. 2, Additional file 2: Figure S2 and Additional file 3: Table S1). The top 20 enriched pathways by each method were also each compared against a benchmark of pathways relevant to psoriasis based on a “psoriasis” keyword query of the Comparative Toxicogenomics Database (CTD) (see Additional file 3: Table S1). In a head to head comparison of each method analysing a psoriatic skin transcriptome (GSE13355), 14/20 pathways identified by cogena were ranked by the CTD benchmark, 13/20 were ranked by WGCNA, 13/20 were ranked by GSEA. Two pathways were common between cogena and WGCNA only, 5 were common between cogena and GSEA only, 3 were common between WGCNA and GSEA only. One pathway, “natural killer cell mediated cytotoxicity” was ranked by all three methods. Despite similar recovery of pathways from the CTD benchmark, qualitatively cogena identified the strongest, disease relevant enrichments in the smaller co-expressed gene clusters, often limited to a single cluster. By contrast WGCNA identified strongest enrichment among a small number of large gene sets, including a cluster of 3568 genes.

Four biologically related groups of pathways, which have an established role in psoriasis, are highlighted in clusters 3, 5, 7 and 9 shown in Fig. 2. Both clusters 3 and 5 are immune-related. In cluster 3, the co-expressed genes are up-regulated and the enriched pathways are consistent with known psoriasis pathology based on the CTD benchmark. The chemokine signaling pathway and cytokine-cytokine receptor interaction pathway, including the IL-23–IL-17 immune axis, play important roles in the pathogenesis of psoriasis and have been targeted by biologic drugs for the treatment of psoriasis with some success [43, 44]. These two pathways were not identified by WGCNA or GSEA. Co-expressed genes in cluster 5 are related to the RIG-I like receptor signalling pathway, cytosolic DNA sensing pathway and toll-like receptor signalling pathway (See Fig. 2). All three pathways are involved in immune response and identified in the CTD psoriasis benchmark, while none is enriched by WGCNA, they are also detected by GSEA. Cluster 7 captures 5 pathways that are broadly related to the cell cycle. Three pathways are also captured in the CTD benchmark, while WGCNA and GSEA only capture one of the five (cell cycle and oocyte meiosis respectively). Though enriched in cogena, CTD and GSEA (ranked 20th, see Additional file 3: Table S1), the oocyte meiosis pathway is likely to represent an overlap with the observed cell cycle enrichment. Both the cell cycle and p53 signaling pathways are highly relevant to the psoriasis, characterized by hyperproliferation and abnormal differentiation of keratinocytes [45]. And the downstream signals of p53 result in apoptosis, senescence and cell cycle arrest. Genes in cluster 9 are down-regulated and show enrichment for the Peroxisome proliferator-activated receptors (PPAR) signaling pathway, only enriched in the results of cogena and CTD. PPAR signalling has an important effect in keratinocyte homeostasis of skin [46]. There are some cases showing the comorbidity of psoriasis and dilated cardiomyopathy, although not Hypertrophic cardiomyopathy (HCM). The calcium signaling pathway was highly ranked by GSEA but not cogena. This may be relevant, as some investigators have speculated that psoriatic keratinocytes may have an inborn error in calcium metabolism [47]. In summary, many more pathways related with psoriasis are discovered by cogena, than by the current state of the art, GSEA and WGCNA methods.

### Drug repositioning based on pathway-guided co-expression analysis

Drug repositioning analysis was performed on the same co-expressed gene clusters analysed by pathway analysis using the clEnrich function based on the CMap gene sets, with the specific aim of aligning pathway and drug mechanism. Furthermore, for the purposes of drug repositioning, the CMap down-regulated gene set should be used to investigate clusters containing up-regulated genes, while the CMap up-regulated gene set should be used for clusters containing down-regulated genes (unless the user is interested in drugs which may induce the disease phenotype under study, perhaps as a side effect). Drug repositioning results generated by cogena were compared with those of CMap and NFFinder webservers using all the DE genes obtained before. As a general accepted approach, the performance comparison is benchmarked using the approved drugs for psoriasis present in the CMap database, resulting in three drugs, Methotrexate, Ciclosporin and Betamethasone [48].

**Fig. 3:**
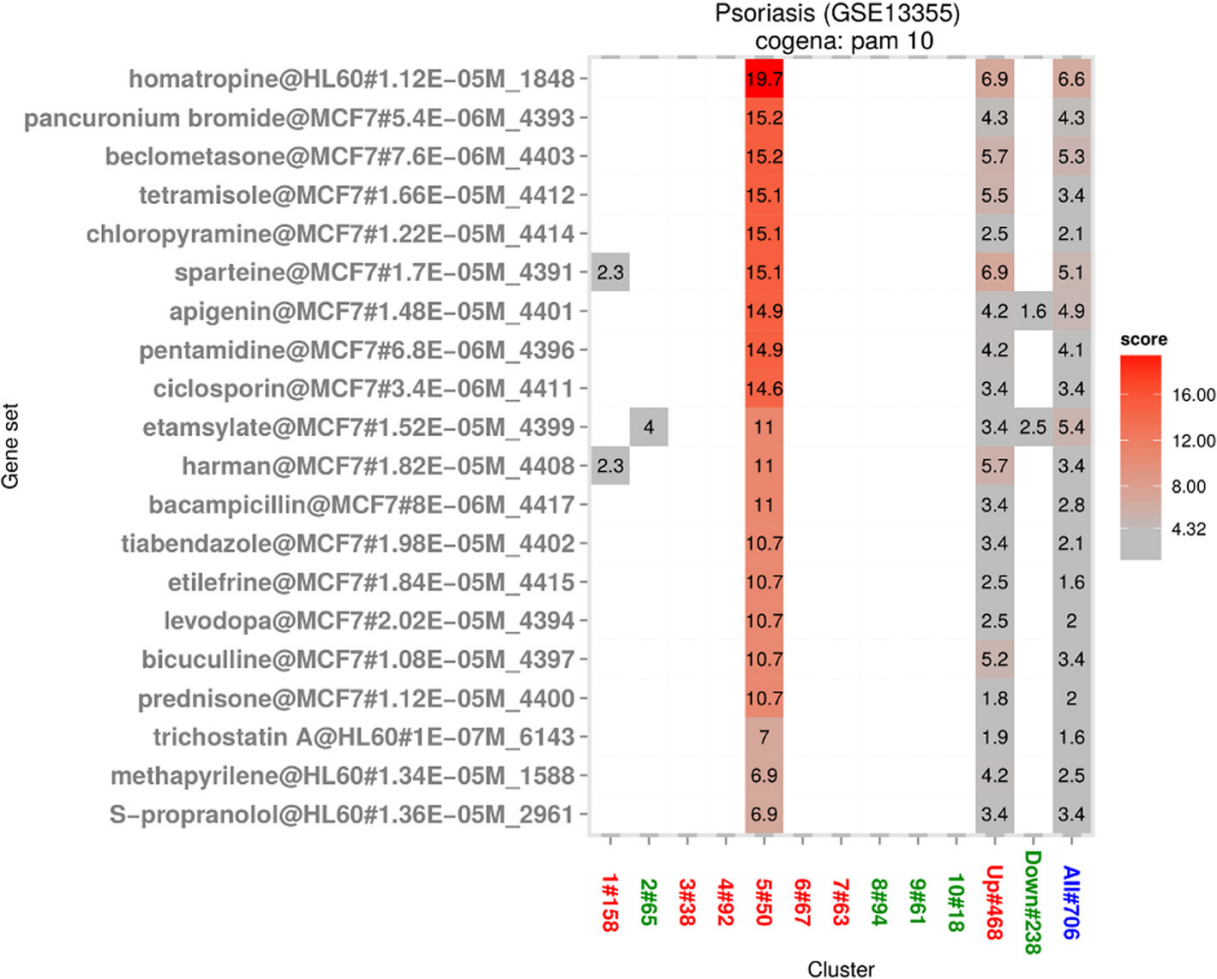
Drug repositioning based on cluster 5 for GSE13355. Enriched drugs with the cell line, dose and instance number are shown on the y axis based on the immune-related cluster obtained by pathway analysis shown in Fig. 2. Ciclosporin, an FDA approved drug for psoriasis, is ranked 9th

We focus our repositioning analysis on clusters 3, 5, 7 and 9, described above. Cluster 3, which is highly enriched for chemokine and cytokine receptor interaction, identifies relatively few drug profile enrichments (See Additional file 4: Figure S3). This may not be surprising considering the historical challenges of drug discovery at chemokine and cytokine receptors [49]. Also it is notable that the pathways represented in cluster 3 are now the focus of biologic therapies in psoriasis [50–52], perhaps in response to the lack of efficacy of small molecule therapies in these pathways. Cluster 5 also showed enrichment for immune related pathways, and by contrast identifies several drugs with known efficacy in psoriasis (See Fig. 3). Two corticosteroids are identified. Beclometasone, ranked 3rd, though efficacious in clearing plaque, is avoided in the treatment of psoriasis due to the risk of rebound on withdrawal. Prednisone, ranked 17th, is an immunosuppressant drug that can clear psoriasis quickly but also with the risk of rebound. An FDA-approved drug for psoriasis, Ciclosporin, is captured and ranked 9th by cogena. Notably, Ciclosporin also has substantial risk of rebound. The 5th drug, Chloropyramine, can be used as a treatment of some allergic conditions, such as allergic conjunctivitis, sharing some clinical similarity with psoriasis. Additionally, Tetramisole, used to treat parasitic worm infections, is an immunomodulator. Drugs identified in cluster 5, for example Beclometasone, Prednisone and Ciclosporin are all effective treatments for psoriasis, but their systemic use has been widely replaced in the clinic by biologics, due to a shared adverse event risk, namely risk of rebound after treatment withdrawal. This emphasises that shared properties of drugs enriched in a given cluster can be used to predict both MoA and possible side effects of a repositioning opportunity. Thus systemic use of drugs identified with action on cluster 5 should probably be avoided, due to risk of rebound, although they could still potentially be used topically.

**Fig. 4:**
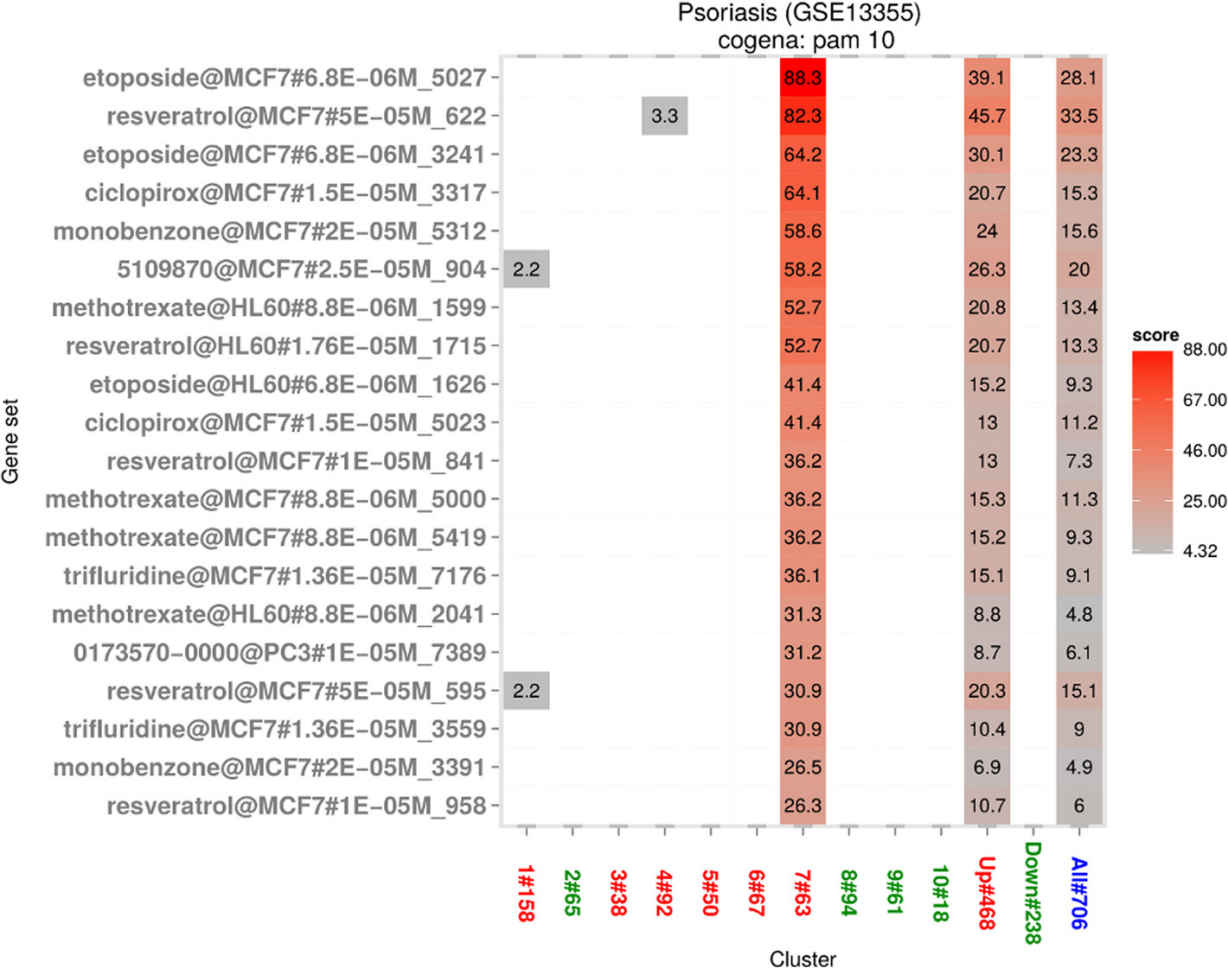
Drug repositioning based on cluster 7 for GSE13355. Enriched drugs with the cell line, dose and instance number are shown on the y axis based on the cell cycle-related cluster obtained by pathway analysis previously. Methotrexate, ranked 7th, is a first-line drug for psoriasis

Thirdly, we investigated CMap drug signatures matching cluster 7, including cell cycle and p53 signaling pathways (See Fig. 4). Some drugs within this cluster have high enrichment scores across multiple instances (representing consistency of action in different cell lines and at different drug doses), suggesting particularly robust association. Methotrexate, a first-line drug for psoriasis, is ranked 7th by cogena. Etoposide is a cytotoxic anticancer drug which belongs to the topoisomerase inhibitor drug class [53]. As a protein synthesis inhibitor, Ciclopirox can decrease DNA replication, protein synthesis, and RNA replication [54] and it is used for topical dermatologic treatment of scalp psoriasis. Monobenzone is used as a topical drug for medical depigmentation. Trifluridine is a nucleoside analogue, whose metabolite inhibits thymidylate synthase and then DNA synthesis [55]. While Methotrexate is in regular clinical use, several of these drugs have previously been considered as candidate drugs for psoriasis, including Resveratrol in the results of CMap analysis as well as in previous works of others [56, 57] and Etoposide [58]. In summary, most of the drugs acting on cluster 7 act at the level of the cell cycle reflecting the pathway enrichment seen for this cluster.

Fourthly, drugs for cluster 9 are analysed (See Additional file 5: Figure S4). Diclofenac is a nonsteroidal anti-inflammatory drug (NSAID), used for the treatment of psoriatic arthritis. As an antineoplastic agent, Mitoxantrone, is a topoisomerase II inhibitor. The types of drugs in this cluster are slightly disordered, though a possible role of PPAR agonists in the management of psoriasis have been suggested [59].

In the results of CMap analysis (See Additional file 6: Table S2), among drugs with negative enrichment scores, Methotrexate is ranked 12th, while Ciclosporin ranked out of top 20 (172th). The ranks of these drugs are relatively lower compared with ranks of drugs in cluster 5 and 7, and importantly it is impossible for CMap itself to link drug to MoA. Concerning the enriched drugs, the results are strikingly consistent with an independent transcriptome-based psoriasis drug repositioning study [57]. In the results of NFFinder based on the CMap database (See Additional file 6: Table S2), both Methotrexate and Ciclosporin are not enriched. In summary, based on the approved drugs for psoriasis comparison, cogena outperformed CMap and NFFinder in this dataset.

### Drawbacks and future development of cogena

There are some drawbacks of the cogena approach, which we are actively seeking to address. Firstly, we are aware that the requirement for manual intervention to select clustering methods and the number of clusters is limiting, and also excludes the possibility of offering cogena as a web service. We also accept that users of cogena benefit from some underlying knowledge of pathways relevant to the disease process under study. The gene sets used by cogena are also important, as they determine not only the type of analysis but also the quality of analysis using cogena. We recognise that there is a need for improved gene sets [60]. As more drug signature data sets become available, we also plan to integrate new data, such as the gene sets abstracted from the new LINCS project. Finally, we emphasise the limitations of computational methods, such as cogena and the genomic data underlying them, in the disclosure of specific drug MOA. However, cogena can certainly highlight pathways which are impacted by drug action, illuminating putative MoA. From this point further studies can be formulated to more specifically investigate drug mechanism in more detail. Additionally, as a flexible framework, cogena can be flexibly applied to many other types of analysis, such as transcription factor and microRNA binding analysis, which we are also investigating.

## Conclusions

Cogena is a fully configurable framework for co-expressed gene set enrichment analysis. We show an application of the cogena framework for pathway analysis, drug repositioning and drug MoA discovery using psoriasis as an exemplar. By combining pathway analysis and drug repositioning analysis, cogena provides a unique approach to imply the drug mode of action in a disease context, which is important to the translational development of computationally repositioned drugs. In conclusion, cogena is a powerful tool for co-expressed gene set enrichment analysis, including pathway analysis and drug repositioning. Cogena is freely available at Bioconductor or https://github.com/zhilongjia/cogena.

### Ethics approval and consent to participate

Not applicable

### Consent for publication

Not applicable

## Availability and requirements

Project name: cogena

Project home page: http://bioconductor.org/packages/cogena/orhttps://github.com/zhilongjia/cogena

Reproducible research: https://github.com/zhilongjia/psoriasis

Operating systems: Platform independent

Programming language: R

Other requirements: None

License: LGPL-3

Any restrictions to use by non-academics: No

## Availability of supporting data

The data set supporting the results of this article is available in the NCBI GEO GSE13355.

The following additional data are available with the online version of this paper. Additional file 1 shows the heatmap of co-expressed genes for GSE13355. Additional file 2 shows the pathway analysis for psoriasis by WGCNA for GSE13355. Additional file 3 is a table listing results of pathway analysis for psoriasis by GSEA, WGCNA and CTD for GSE13355. Additional file 4 is a figure showing the results of drug repositioning based on cluster 3 for GSE13355. Additional file 5 is a figure showing the results of drug repositioning based on cluster 9 for GSE13355. Additional file 6 details results of drug repositioning for psoriasis based on CMap and NFFinder for GSE13355.

## Additional files

Additional file 1: Figure S1. Heatmap of co-expressed genes for GSE13355. The co-expressed genes are clustered by the PAM method with 10 clusters and shown in the left bar. The up-regulated and down-regulated genes are also indicated in the far left bar. (PNG 663 kb) Additional file 2: Figure S2. Results of pathway analysis for psoriasis by WGCNA for GSE13355. Pathways results based on the co-expression genes obtained WGCNA. (PNG 442 kb) Additional file 3: Table S1. Results of pathway analysis for psoriasis by GSEA, WGCNA and CTD for GSE13355. Pathways from the result of GSEA and pathways in CTD with “psoriasis” as the enquiry are shown in this file, respectively. (XLS 82 kb) Additional file 4: Figure S3. Drug repositioning based on cluster 3 for GSE13355. Enriched drugs with the cell line, dose and instance number are shown on the y axis based on the immune-related cluster obtained by pathway analysis before. (PNG 74 kb) Additional file 5: Figure S4. Drug repositioning based on cluster 9 for GSE13355. Enriched drugs with the cell line, dose and instance number are shown on the y axis based on the PPAR signaling-related cluster obtained by pathway analysis before. (PNG 241 kb) Additional file 6: Table S2. Results of drug repositioning for psoriasis based on CMap and NFFinder for GSE13355. The drug repositioning results by CMap and NFFinder are shown. (XLS 972 kb)

## Abbreviations

CMap: connectivity map
Cogena: co-expressed gene-set enrichment analysis
CTD: comparative toxicogenomics database
DE: differentially expressed
GSEA: gene set enrichment analysis
LINCS: library of integrated cellular signatures
MoA: mode of action
MOA: mechanism of action
WGCNA: weighted correlation network analysis
