# Supplementary figures and images for: Cogena, a novel tool for co-expressed gene-set enrichment analysis, applied to drug repositioning and drug mode of action discovery
Source: BMC Genomics. 2016 May 27;17:414. doi: 10.1186/s12864-016-2737-8 (PMC4884357; doi:10.1186/s12864-016-2737-8)

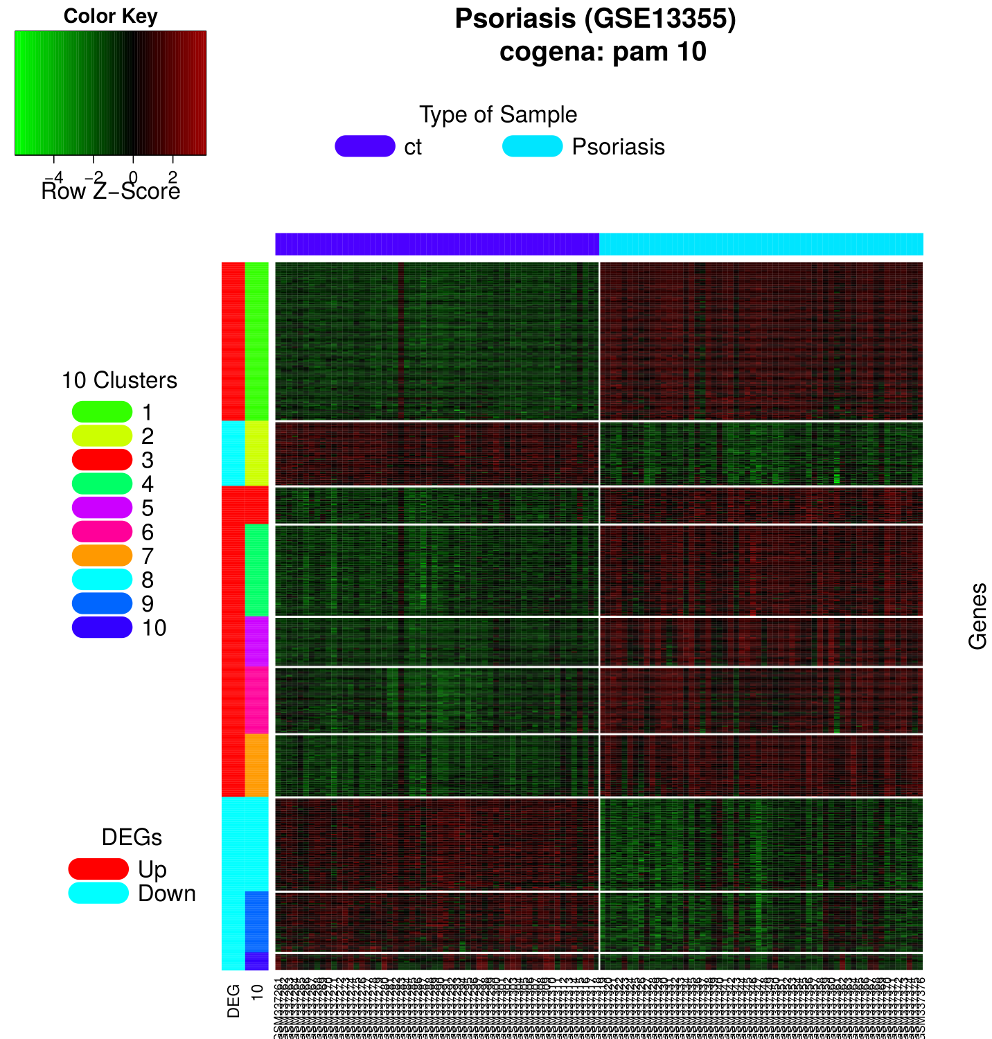

Supplement: Additional file 1: Figure S1. — Heatmap of co-expressed genes for GSE13355. The co-expressed genes are clustered by the PAM method with 10 clusters and shown in the left bar. The up-regulated and down-regulated genes are also indicated in the far left bar. (PNG 663 kb) [file 12864_2016_2737_MOESM1_ESM.png]

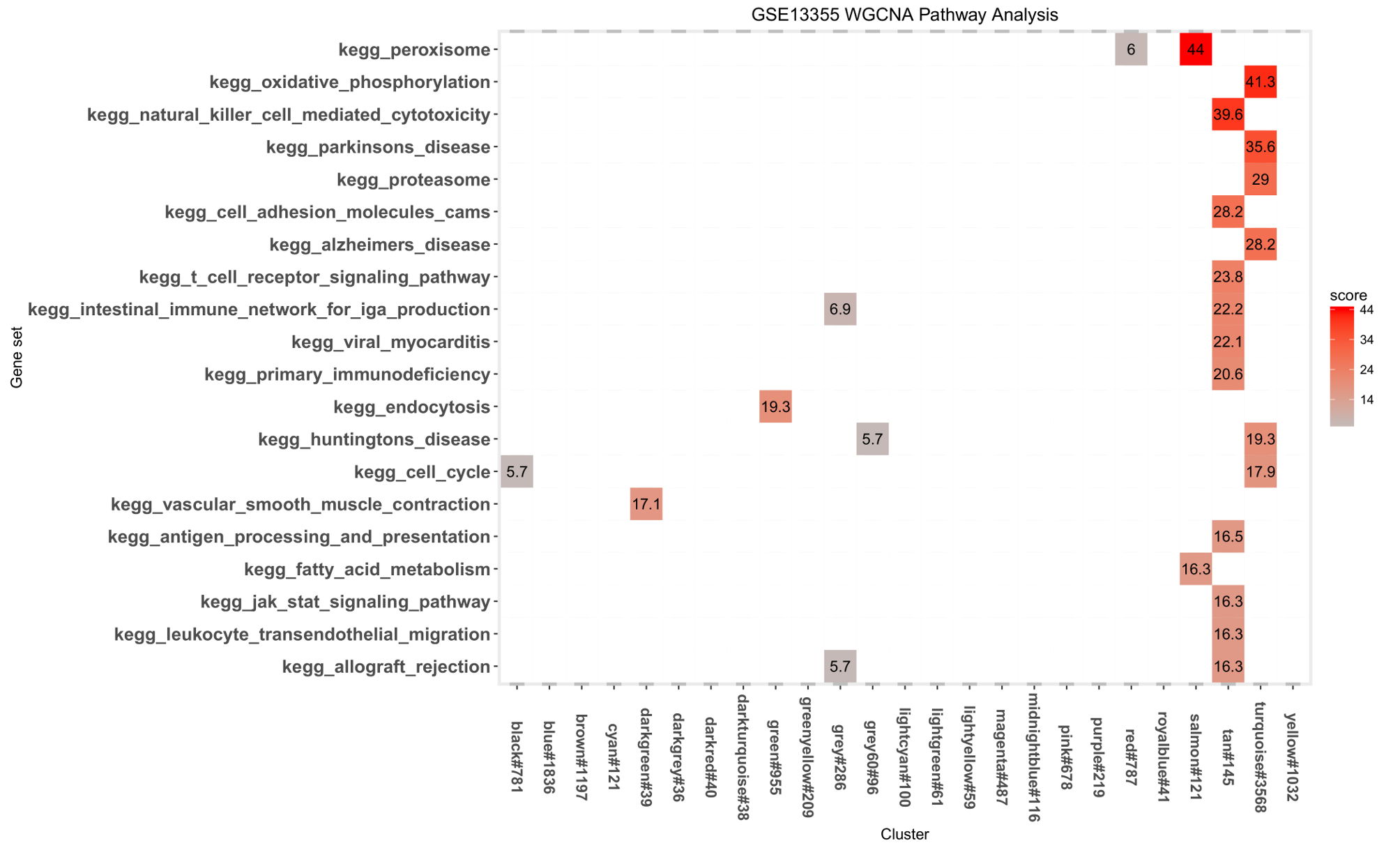

Supplement: Additional file 2: Figure S2. — Results of pathway analysis for psoriasis by WGCNA for GSE13355. Pathways results based on the co-expression genes obtained WGCNA. (PNG 442 kb) [file 12864_2016_2737_MOESM2_ESM.png]

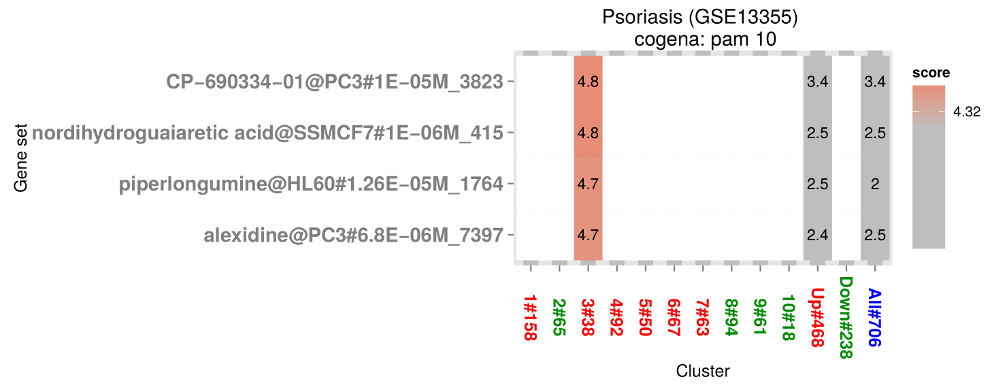

Supplement: Additional file 4: Figure S3. — Drug repositioning based on cluster 3 for GSE13355. Enriched drugs with the cell line, dose and instance number are shown on the y axis based on the immune-related cluster obtained by pathway analysis before. (PNG 74 kb) [file 12864_2016_2737_MOESM4_ESM.png]

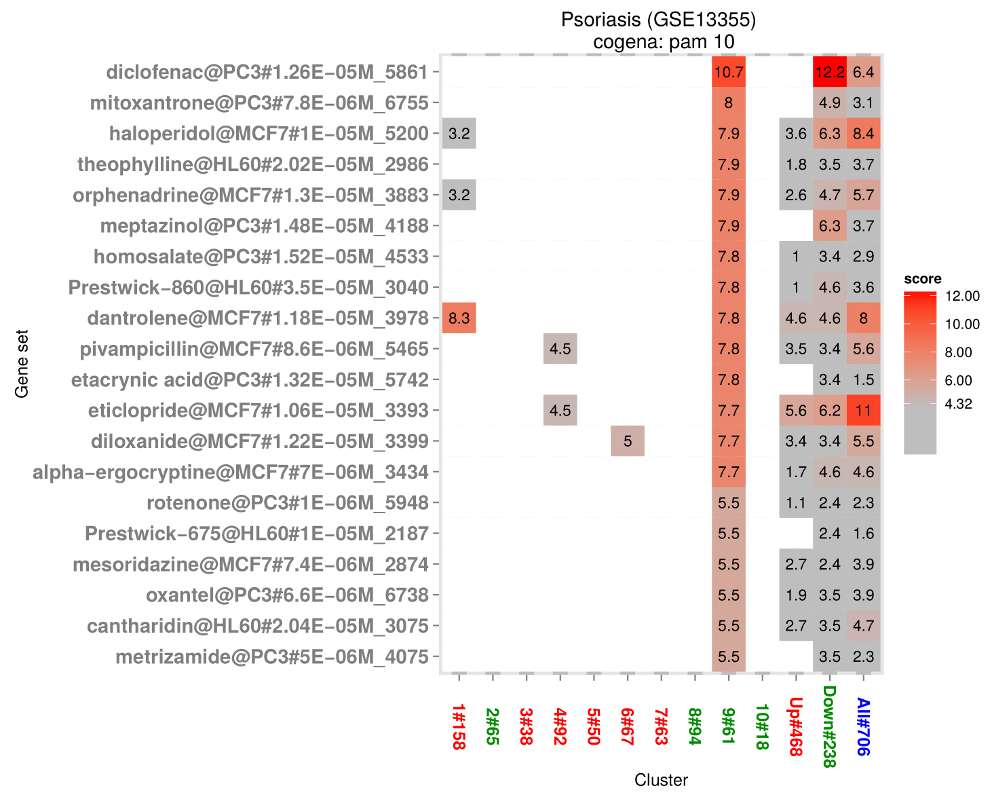

Supplement: Additional file 5: Figure S4. — Drug repositioning based on cluster 9 for GSE13355. Enriched drugs with the cell line, dose and instance number are shown on the y axis based on the PPAR signaling-related cluster obtained by pathway analysis before. (PNG 241 kb) [file 12864_2016_2737_MOESM5_ESM.png]
